# Decellularized liver scaffolds promote liver regeneration after partial hepatectomy

**DOI:** 10.1038/s41598-019-48948-x

**Published:** 2019-08-29

**Authors:** Hirofumi Shimoda, Hiroshi Yagi, Hisanobu Higashi, Kazuki Tajima, Kohei Kuroda, Yuta Abe, Minoru Kitago, Masahiro Shinoda, Yuko Kitagawa

**Affiliations:** Department of Surgery, Keio University, School of Medicine, Tokyo, Japan

**Keywords:** Liver, Biomedical materials

## Abstract

The resectable liver volume is strictly limited and this reduces the number of patients who may be treated. Recently, “tissue/organ decellularization”, a new approach in bioengineering, has been investigated for its ability to produce a native organ scaffold by removing all the viable cells. Such a scaffold may support the repair of damaged or injured tissue. The purpose of this study was to evaluate the potential contribution of liver scaffolds to hepatic regeneration after hepatectomy. We sutured the partial liver scaffolds onto the surfaces of partially hepatectomized porcine livers and assessed their therapeutic potential by immune histological analysis at various time points. Animals were sacrificed after surgery and the implanted scaffolds were evaluated for the infiltration of various types of cells. Immune histochemical study showed that blood vessel-like structures, covered with CD31 positive endothelial cells and ALB positive cells, were present in all parts of the scaffolds at days 10 and 28. Blood inflow was observed in some of these ductal structures. More interestingly, CK19 and EpCAM positive cells appeared at day 10. These results suggest that the implantation of a decellularized organ scaffold could promote structural reorganization after liver resection.

## Introduction

Hepatic resection is a useful radical treatment for various liver tumors; however, there is a strict limit to the resectable liver volume^1,2^ which reduces the number of patients who may be treated. Recent progress in tissue regeneration technology may increase this limit and minimize the risk of post-operative liver failure.

Interest in one of these regeneration technologies, decellularization, has increased significantly. The removal of the cells from an organ leaves a complex mixture of structural and functional proteins that constitute the extracellular matrix (ECM)^3^. Several studies^4–6^ have shown that this microenvironment plays a fundamental role, not only in cell maintenance and homeostasis, but also in determining stem cell fate^7^. The ECM and its three dimensional structure are essential components of this microenvironment and have been exploited for the maintenance of somatic cells, cancer cells and stem cells *in vitro*, using tissue engineering approaches, demonstrating supportive activity in cell cultures^8^. Therefore, ECM technology will be highly beneficial in combination with stem cell biology for the further improvement of regenerative therapy.

Recent studies have shown that decellularization of whole organs, such as kidney, liver, lung and heart, are possible in animal models^9–11^. Particularly, our group demonstrated that this decellularization technology could be applied to a large animal model^12^. The decellularization process preserves the functional characteristics of the native microvascular and bile drainage networks of the liver and the growth factors necessary for angiogenesis and liver regeneration. Thus, it is feasible that ECM scaffolds could be used to promote liver regeneration by enabling macroscopic regrowth into a damaged liver.

The purpose of this study was to engineer liver-derived ECM scaffolds capable of inducing liver regeneration after partial hepatectomy. We hypothesized that, by providing a neutral environment that mimics normal physiological conditions, the ECM scaffolds would allow various types of liver cells to infiltrate or migrate into them from the residual liver.

## Results

### Characterization of DC liver scaffolds

**Figure 1:**
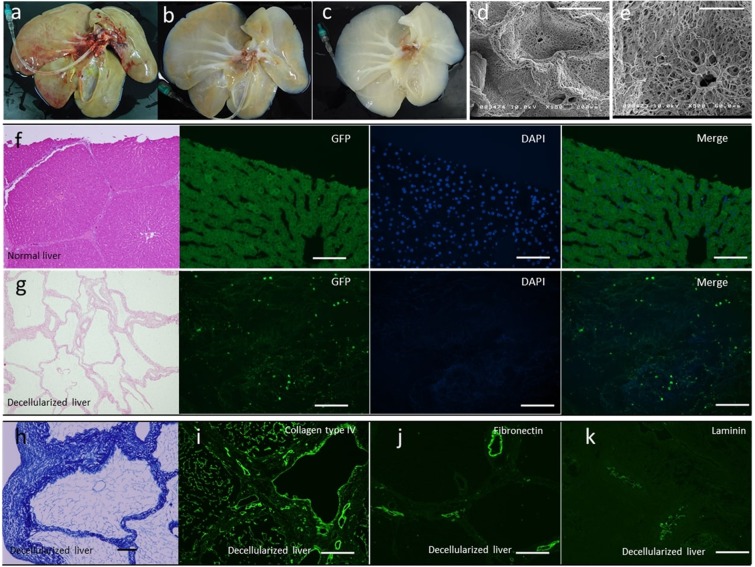
Characterization of porcine liver scaffolds. The decellularization process with continuous detergent perfusion at (**a**) 0 h, (**b**) 48 h and (**c**) 96 h. (**d**,**e**) Ultrastructural characterization of the decellularized liver matrix; structures such as the hepatic lobules, the central veins, portal triad, and extracellular matrix are present within the parenchyma. (**f**,**g**) The presence of intact nuclear material was evaluated by staining the decellularized liver and native liver with hematoxylin and eosin and 4¢,6-diamidino-2-phenylindole (DAPI). (**h**) Azan staining of decellularized porcine liver scaffolds. Immune-histochemical staining of decellularized porcine liver scaffolds for collagen IV (**i**), fibronectin (**j**), and laminin (**k**). Scale bars: 100 µm.

The protocol for whole organ decellularization was based on the work of Yagi *et al*.^12^. Figure 1a–c shows the decellularization process with continuous detergent perfusion, which generated acellular scaffolds of porcine liver. H&E staining revealed that blue-stained nuclei were not detectable but pink-stained components were present in the liver scaffold (Fig. 1g), compared with the control (Fig. 1f). Because the pink-stained components include both cytoplasm and extracellular matrix, the morphological difference in H&E staining between intact liver and DC liver scaffolds is quite clear. In addition, DAPI staining (Fig. 1g) showed no visible nuclear material in the decellularized liver matrix. Azan staining was used to examine the collagen fibers and revealed that positively stained structures were present in the DC liver scaffolds as small lobular components (Fig. 1h). Furthermore, immunostaining of extracellular matrix proteins, collagen type IV (Fig. 1i), fibronectin (Fig. 1j) and laminin (Fig. 1k), indicated that the structural and basement membrane components of the ECM were retained, similar to native liver. Finally, scanning electron microscopy (SEM) was performed for the ultrastructural characterization of the decellularized liver matrix and confirmed the presence of structures including the hepatic lobules, the central veins, portal triad, and extracellular matrix within the parenchyma (Fig. 1d,e).

### The scaffold promoted regeneration of ductal structure and liver lobe after liver resection

**Figure 2:**
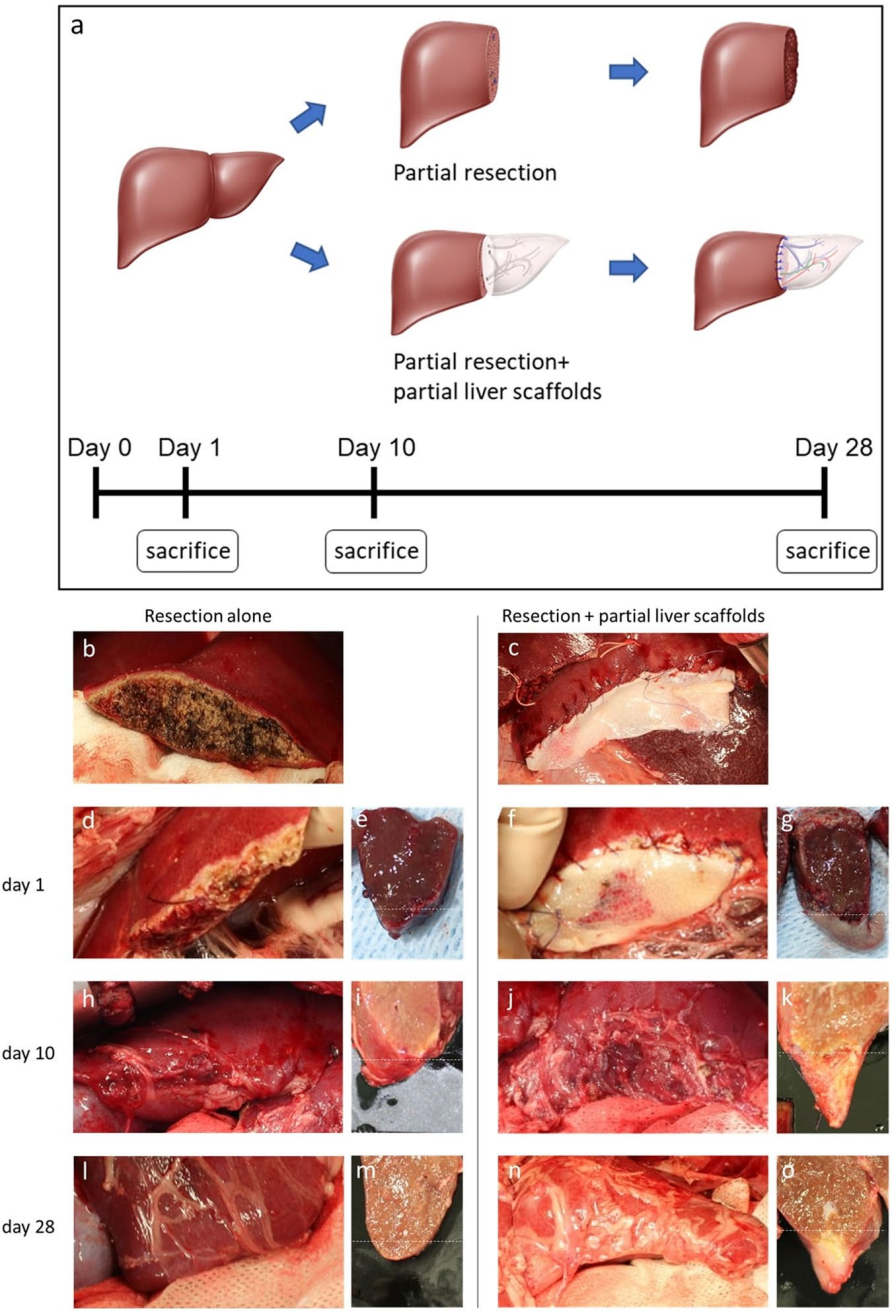
The porcine model of liver regeneration with liver-derived ECM scaffolds. (**a**) Showing the procedures of grafting DC liver scaffolds. Control lobes were partially hepatectomized without suturing any scaffolds. Illustration by Yoshihiko Tsuda. Printed with permission by Yoshihiko Tsuda under a CC BY open access license (https://creativecommons.org/licenses/by/4.0/) (**b**), whereas, for the treatment lobes, the partial liver scaffolds were sutured onto the surfaces of similarly sized, partially hepatectomized porcine livers, completely covering the cut area (**c**). Animals were sacrificed 1, 10 and 28 days after surgery. (**h**,**j**,**l**,**n**) Mild adhesion to the peritoneum and omentum was observed around the resection or sutured scaffold site on days 10 and 28 after surgery. (**f**,**g**) The scaffolds retained their original structural features and the borderline between the native liver and the scaffold was clearly visible macroscopically. (**j**,**k**) The borderline had become less clear and macroscopic regrowth of liver tissue into the sutured acellular scaffold was seen. (**n**,**o**) Macroscopically, the volume of the sutured scaffold looked smaller and the parenchymal space of the scaffold had become slightly aggregated.

The potential for hepatic regeneration using these engineered scaffolds was examined by suturing them onto partially hepatectomized porcine livers (Fig. 2a–c). At day 1, the scaffolds retained their original structural features and the borderline between the native liver and the scaffold was clearly visible macroscopically (Fig. 2g). Macroscopic evaluation of specimens collected on days 10 and 28 after surgery revealed mild adhesion to the peritoneum and omentum around the resection or sutured scaffold site (Fig. 2h,j,l,n). By day 10, the borderline had become unclear (Fig. 2k) and macroscopic regrowth of liver tissue into the sutured acellular scaffold was seen in the experimental tissue (Fig. 2k). The macroscopic volume of the sutured scaffold looked smaller and the parenchymal space of the scaffold had become slightly aggregated (Fig. 2k). By 28 days, this trend had become more noticeable (Fig. 2o).

**Figure 3:**
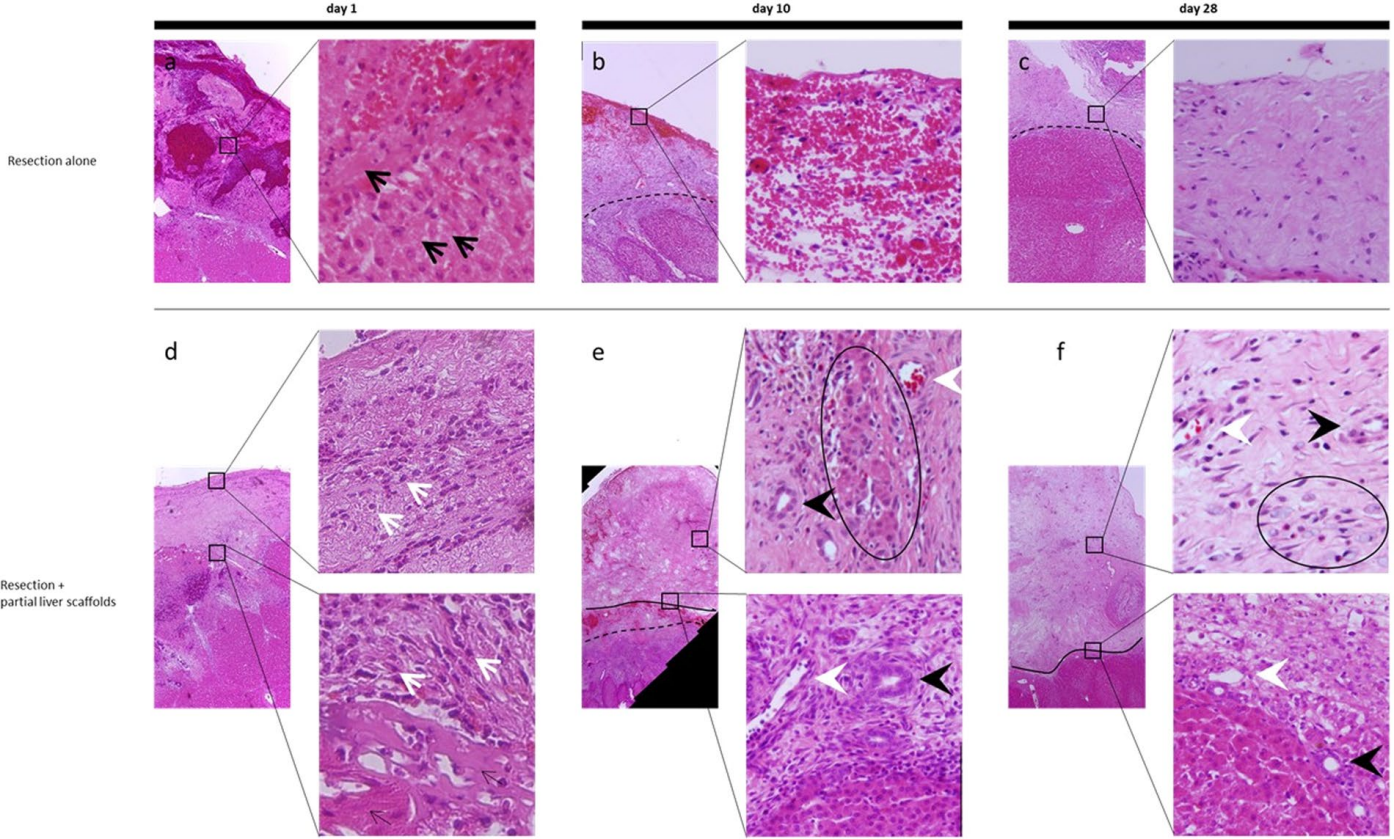
Histological analysis at 1, 10 and 28 days after surgery. (**a**) Only coagulation and enucleated cells (black arrows) were seen. (**b**,**c**) The tissue from the control experiment at days 10 and 28 showed only hyalinized hepatocytes and inflammation following necrosis. The black dotted line represents the border between the injured areas and intact areas. (**d**) Infiltration of inflammatory cells and fibroblastic cells (white arrows) was observable in the tissue samples of the scaffolds. (**e**) Blood vessel-like structures (white arrowheads), bile duct-like cavities (black arrowheads) and clumps of liver parenchymal cells (indicated as circle) were observed throughout the sutured scaffold. (**f**) Blood-vessel-like structures were seen more clearly. Bile duct-like cavities and clumps of liver parenchymal cells also could be observed at 28 days. The black solid line represents the border between the liver parenchyma or injured areas and the scaffolds.

Although, H&E staining showed that infiltration of inflammatory cells and fibroblastic cells was observable throughout the tissue samples of the scaffolds of the experimental lobe on day 1 (Fig. 3d), this had reduced at day 10, while blood vessel-like structures were observed throughout the sutured scaffold (Fig. 3e). Finally, after long-term observation at 28 days, blood-vessel-like structures were seen more clearly (Fig. 3f), compared to the tissues from day 1, which had shown only coagulation and enucleated cells (Fig. 3a), and the tissue from the control experiment at days 10 and 28, which showed only hyalinized hepatocytes and inflammation following necrosis (Fig. 3b,c). Furthermore, fibrous areas or granulation tissues, which were observed at the resection surface of the control porcine livers, narrowed on day 10 and rarely seen on day 28 at the borderline between the native organ and the sutured acellular scaffold (Fig. 3b,c,e,f).

### Vascular structures and blood flow were recognized in the decellularized liver scaffold, which consisted of CD31-positive endothelial cells

**Figure 4:**
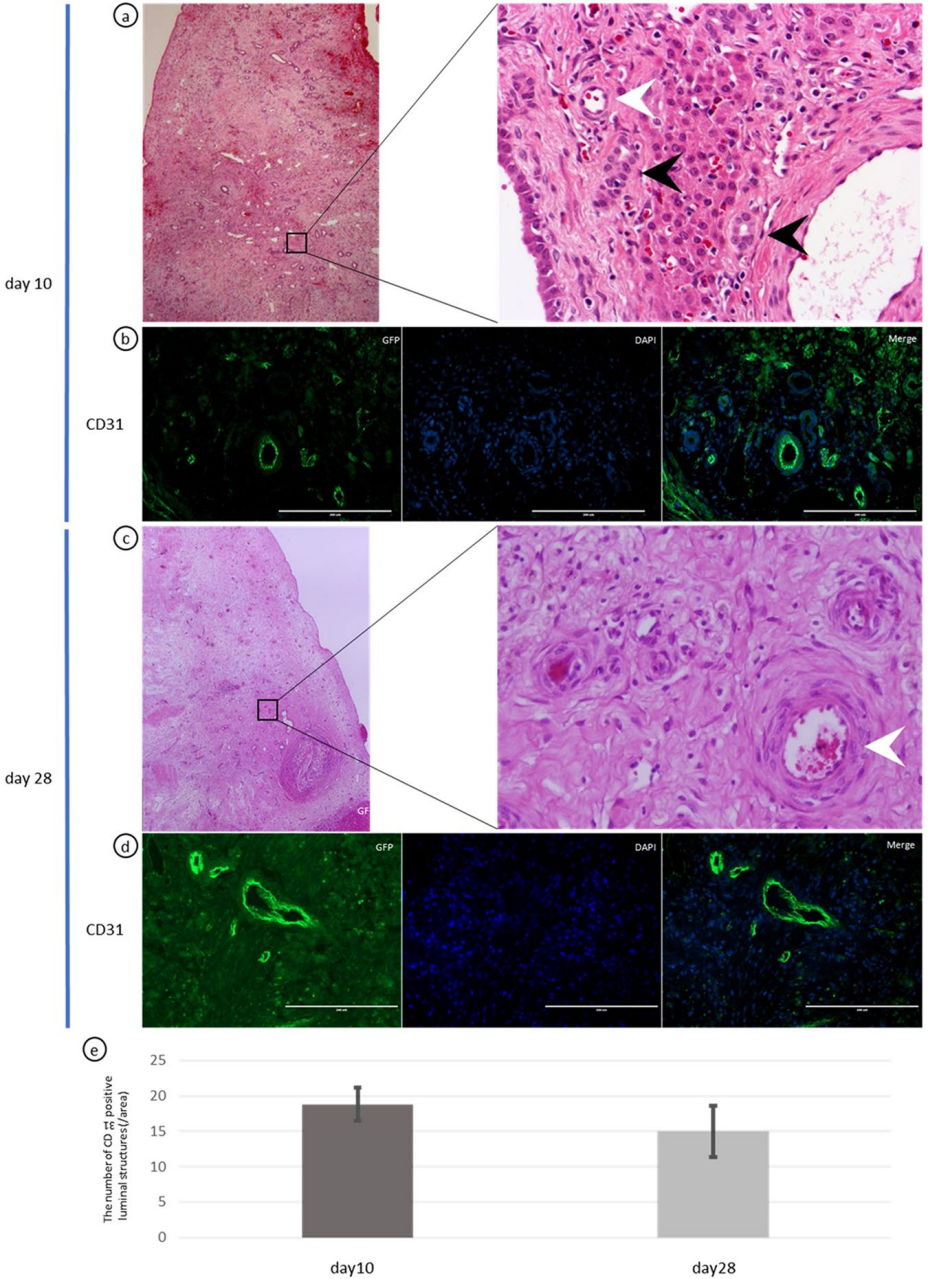
Vascular structures and blood flow in the decellularized liver scaffold. Immune-histochemical evaluation of CD31-positive endothelial cells. (**a**,**c**) Infiltration of red blood cells into some of the blood vessel-like components (white arrow) and luminal structures, composed of one layer of epithelial cells, were observed in some parts of the sutured scaffold (black arrow) on day 10. (**b**,**d**) CD31-positive cells with blood vessel-like structures were seen throughout broad areas in the sutured scaffold on days 10 and 28. (**e**) There was no significant difference in the numbers of CD 31-positive luminal structures on days 10 and 28. Scale bars: 200 µm. p > 0.05.

**Figure 5:**
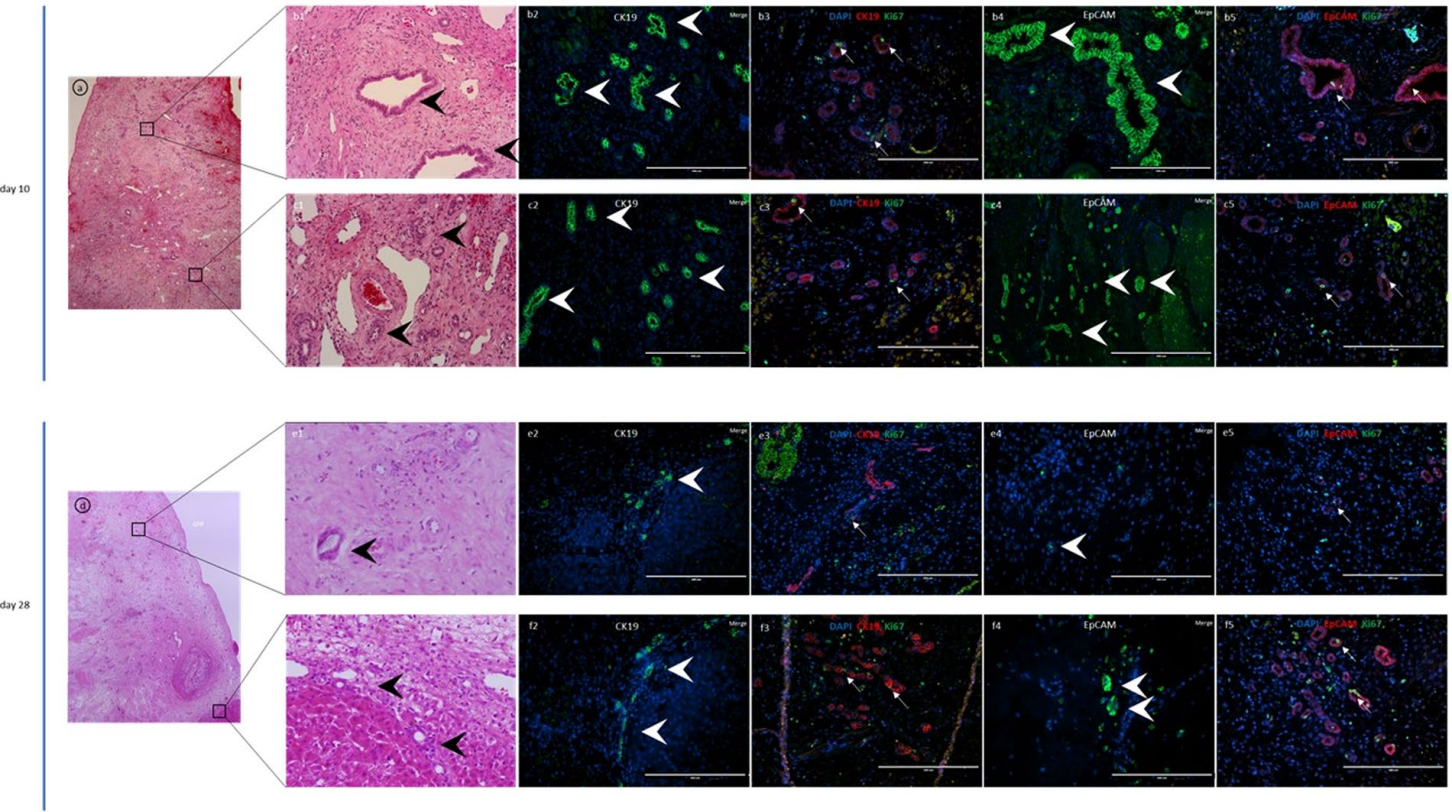
CK19- and EpCAM-positive cholangiocytes in the decellularized liver scaffold. (**a**, b1, c1) Luminal structures, composed of one layer of epithelial cells, were observed in some parts of the sutured scaffold. (b2-5, c2-5) CK19- and EpCAM-positive cells were present as recovering, bile duct-like structures, close to the resected margin on day 10. Some of those cells showed Ki67 positive, by double staining. (**d**, e1, f1) The bile duct-like cavities had decreased inside the liver scaffold but were still observed at the borderline between scaffold and native liver. (e2-5, f2-5) CK19- and EpCAM-positive cells were not clearly observed in the scaffolds, although they remained near the resected margin on day 28 and some of them were Ki67 positive. Scale bars: 200 µm.

**Figure 6:**
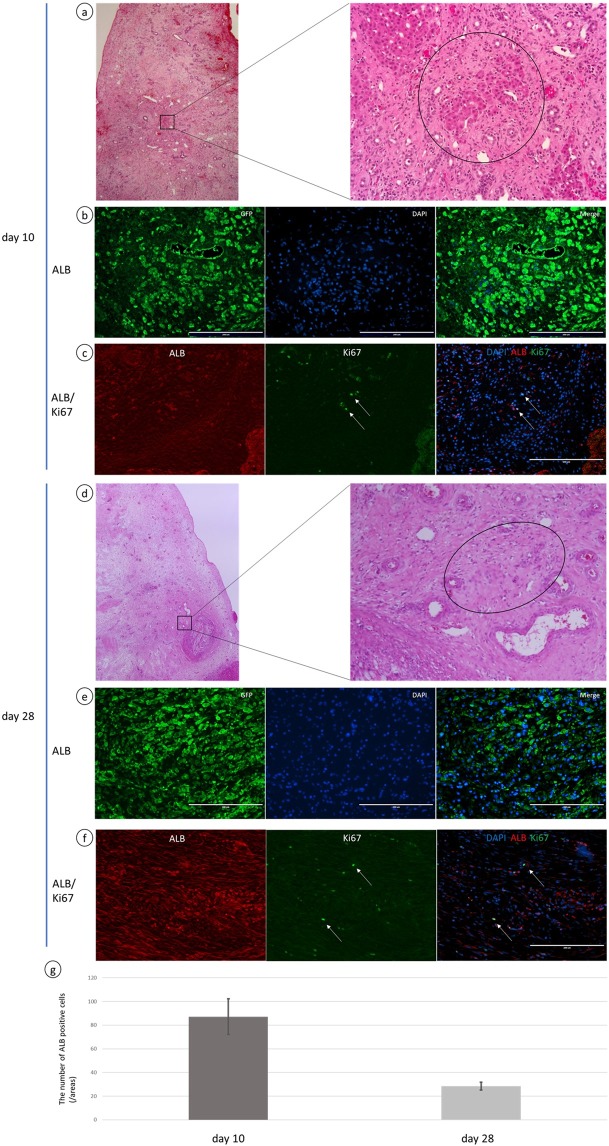
Hepatocytes had infiltrated into the scaffold. (**a**,**d**) Part of the liver scaffold had been repopulated by parenchymal cells. (**b**,**e**) ALB positive cells were observed on days 10 and 28. The parenchymal cells observed by H&E staining were ALB positive. (**c**,**f**) A few Ki67-positive cells were present on days 10 and 28 and double staining of Ki67/ALB revealed some of the ALB positive cells were ki67- positive. (**g**) The number of ALB positive cells was greater on day 10 than on day 28. Scale bars: 200 µm. p < 0.05.

Interestingly, microscopic examination revealed infiltration of red blood cells into some of the blood vessel-like components (Fig. 4a white arrowhead). More importantly, luminal structures, composed of one layer of epithelial cells, were observed in some parts of the sutured scaffold (Fig. 5a,b1,c1, black arrowhead), suggesting bile duct regeneration. On day 28, a large number of red blood cells was observed in these cavities (Fig. 4c white arrowhead), however, the bile duct-like cavities, which were seen on day 10, had decreased inside the liver scaffold, but were still observed at the borderline between scaffold and native liver (Fig. 5d,e1,f1, black arrowhead). In addition, part of the liver scaffold had been repopulated by parenchymal cells, which resembled hepatic-lobule like structures, on days 10 and 28 (Fig. 6a,d).

To evaluate the distribution and types of cells that had infiltrated or migrated into the scaffold, immune histochemical analysis was performed on days 10 and 28. CD31-positive cells with blood vessel-like structures were seen throughout broad areas in the sutured scaffold on days 10 and 28 after the implantation (Fig. 4b,d). There was no significant difference in the numbers of CD 31-positive luminal structures on days 10 and 28 (Fig. 4e).

### CK19- and EpCAM-positive cholangiocytes and hepatocytes had infiltrated into the scaffold

In addition, expression of albumin as a marker of hepatocytes, CK19 for cholangiocytes, EpCAM for hepatic progenitor cells and Ki67 for the proliferation of hepatocytes were examined (Figs 5, 6). CK19 and EpCAM positive cells were present as recovering bile duct-like structures, throughout the scaffold from close to the resected margin on day 10 (Fig. 5b,c) but they were rarely observed in the scaffolds, although they remained near the resected margin on day 28 (Fig. 5e,f). Interestingly, some of those cells showed Ki67 positive, by double immunostaining (Fig. 5b3,b5,c3,c5,e3,e5,f3,f5). On the contrary, ALB positive cells were observed on days 10 and 28 (Fig. 6b,e) but the number of ALB positive cells was greater on day 10 than on day 28 (Fig. 6g). Parenchymal cells observed by H&E staining were ALB positive, but CK19- and EpiCAM-positive cells were not present (Figs 5b,c,e,f, 6b,e). Double immunostaining of Ki67/ALB revealed some of the ALB positive cells were ki67- positive on days 10 and 28 (Fig. 6c,f).

### Comparison of gene expression between healthy liver and scaffold 10 or 28 days after implantation

**Figure 7:**
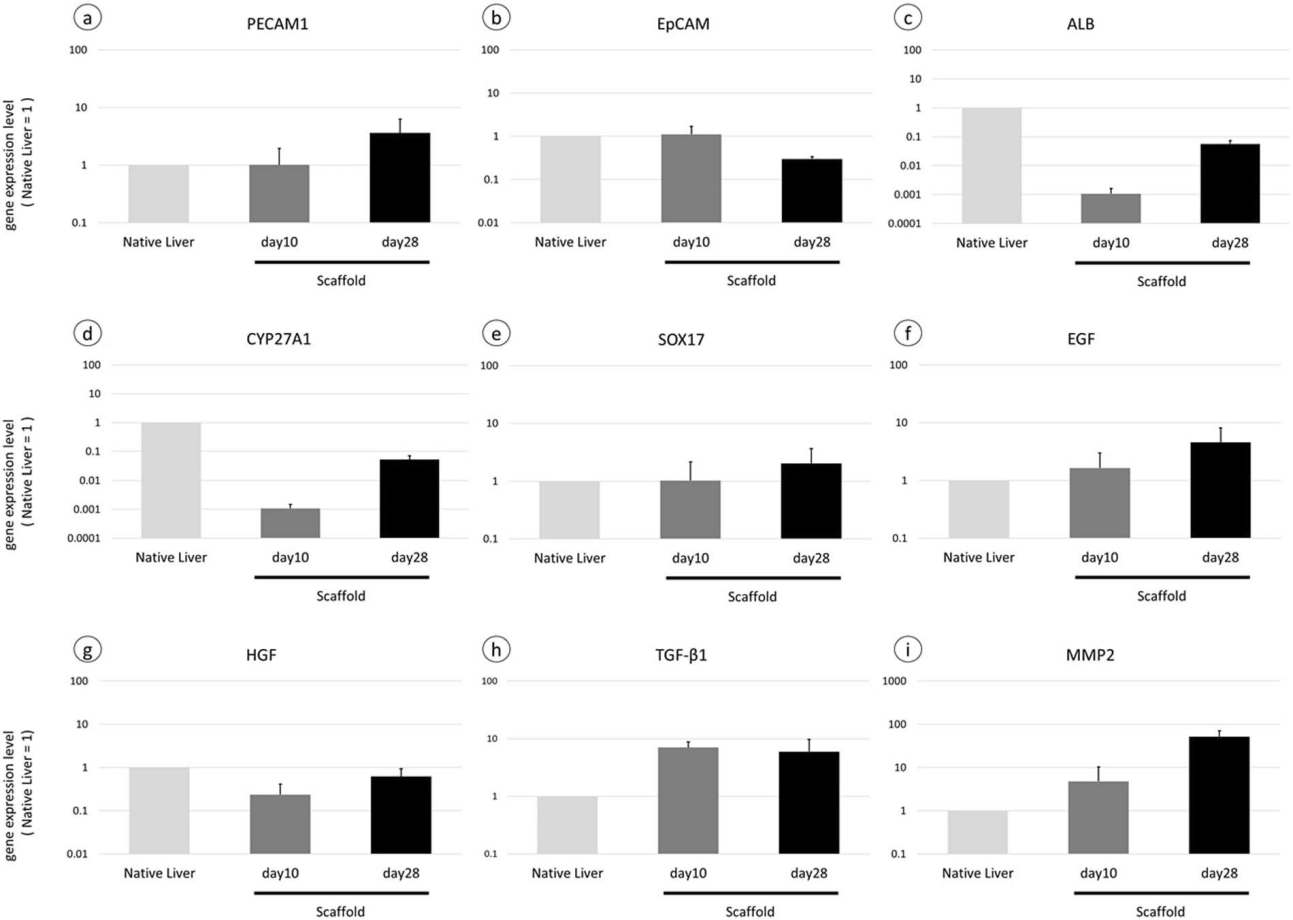
Real-time RT-PCR analysis of 3 samples (healthy liver, sutured scaffold 10 days after implantation and sutured scaffold 28 days after implantation). Gene expression of (**a**) PECAM1, (**b**) EpCAM, (**c**) ALB, (**d**) CYP27A1, (**e**) SOX17, (**f**) EGF, (**g**) HGF, (**h**) TGFβ1, and (**i**) MMP2 was observed in sutured scaffold. Bars represent the mean ± SEM (n = 3).

For further analysis of the functional regeneration in the liver scaffold, we assessed gene expression of cells which appeared in scaffolds in this model. The relative gene expression level of PECAM1 and EpCAM in the scaffolds was as well as in the healthy liver on days 10, and gene expression of EpCAM decreased while PECAM further increased on days 28 (Fig. 7a,b). These results matched with those of immunostaining. On the other hands, compared with healthy liver, there are quite a few gene expression of ALB and CYP27A1 on days 10, however relatively higher gene expression was observed on days 28 (Fig. 7c,d), suggesting that hepatocytes with metabolic capacity appeared in scaffold and increased with time. Furthermore, gene expression of endoderm‐specific marker SOX17 was observed at similar level as healthy liver (Fig. 7e), and gene expression related to promotion and suppression of liver regeneration such as EGF, HGF, TGFβwas also observed in the scaffold on days 10 and 28 (Fig. 7f–h). Finally, MMP2 expression was higher in the scaffold than healthy liver and more upregulated on days 28 (Fig. 7i), suggesting that remodeling of extracellular matrix is activated in the scaffold.

## Discussion

The ECM is composed predominantly of protein and retains various growth factors, which influence all basic functions, including proliferation, stabilization and differentiation of the surrounding cells^13^. From the early stage of fetal development, the ECM plays a central role by mediating biophysical stimuli, biochemical and molecular signals, and spatial organization^6,14^. In particular, the continuous and dynamic interrelationship between cells and the ECM contributes to the conversion from cell proliferation to structure formation^5,15^. A recent study shows that the three dimensional structure itself, formed by the ECM, greatly affects the capacity of stem cells and other cells for regeneration; furthermore, the decellularized liver scaffold provides microvascular networks for oxygen and nutrient transport, as well as metabolite excretion^16^. Finally, the decellularized organ scaffold has low antigenicity^17^; therefore, the use of this technology for therapeutic applications has a great advantage.

With recent advances in liver tissue engineering, attempts have been made to make artificial liver grafts by decellularization and reseeding with primary hepatocytes, with subsequent perfusion of the cells^18–20^. We considered that the decellularized liver scaffold itself contains sufficient extracellular matrix to induce the proliferation and differentiation of liver progenitor cells; therefore, the scaffold alone might have potential for liver regeneration. This is the first attempt to use decellularized liver scaffolds after hepatectomy and to determine whether the acellular scaffolds facilitate liver regeneration in partially hepatectomized animals, using a large model. The results of this study were that the scaffold alone, without recellularization, may help the repair of damaged or injured tissue in terms of liver regeneration after hepatectomy.

Yu *et al*. reported that a rat-derived kidney ECM scaffold, sutured onto the resection surface of a partially nephrectomized rat kidney, can induce macroscopic growth and regeneration of damaged kidneys, with function recovery^21^. In our study, a similar process was carried out using porcine livers.Vasculature and liver parenchymal cells were present from an early stage; however, maintenance of the parenchymal cells was not observed in the long-term. This differs from the renal regeneration with kidney scaffolds in the rat model.

In the early period of the liver regeneration, the damaged site contains necrotic hepatocytes with infiltrating neutrophils, monocytes and macrophages^22^. In particular, macrophages are derived from monocytes and Kupffer cells and these macrophages induce granulomas^23^. After a few days, reticulin fibers increase progressively from the periphery of the damaged area and extracellular matrix, including type I and IV collagen, as well as laminin, begins to increase. At the end of this period, necrotic areas are replaced by granulation tissue and the interstitial fibrotic tissue gradually condenses. Finally, the granulation tissue disappears, being replaced by normal hepatic tissue^22,24,25^.

It seems, in this study, that this process occurred at the resection surface in the control lobes and progressed to fibrosis. However, when we adapted the shape of the liver-derived organ scaffold to the resection surface of the native organ in the animals, and sutured the capsule of this scaffold, various types of the cells, including ALB, CD31, CK19 and EpCAM positive cells, had infiltrated into the scaffold from the native organ, even on day 10, compared to the control without the scaffold.

During the regeneration process at 28 days, neovascularization had occurred in all areas of the scaffold, with internal blood flow. We think the reason for this phenomenon is that the presence of microvascular networks of liver scaffold may contribute to the engraftment of endothelial cells and vascular remodeling. In addition, in the process of regeneration, at day 10, bile duct structures, showing EpCAM positivity stronger than the native liver, also appeared in the scaffolds; moreover, clumps of hepatocytes were seen around the vasculature up to day 28. These results suggest that the revascularization in the scaffolds caused a periportal ductular reaction and hepatic regeneration might be promoted^26^.

Furthermore, fibrous areas or granulation tissues, which were generally observed at the resection surface of the control pig livers, were rarely seen at the borderline between the native organ and the sutured acellular scaffold. This suggests that the sutured scaffolds might facilitate the regeneration of the liver, without inducing fibrosis at the resection surface. This, in turn, may be due to suppression of activation of macrophages or stellate cells. Indeed, stellate cells have been reported to produce the extracellular matrix which is necessary for liver regeneration and, conversely, induce liver fibrosis that could decrease the liver’s inherent regenerative capacity^27^. Therefore, the presence of liver derived organ scaffolds which consisted of overall extracellular matrix may inhibit the activation of stellate cells and macrophages which may play an important role in the regenerative ability of this engineered natural scaffold in the early stage of liver regeneration. The results from microscopic imaging revealed that ALB-positive hepatocytes could be migrating into the sutured liver-derived organ scaffold from the injured liver, suggesting that the intact ECM structure of the natural liver scaffold retained sufficient chemical and/or physical signals to induce the proliferation and differentiation of liver progenitor cells, present nearby. These results suggest that the implantation of liver derived organ scaffolds can promote the structural reorganization after the liver resection, even at the early time points.

However, on day 28, CK19 and EpCAM positive cells were still observed at the borderline between the native liver and the scaffold but were less visible in the scaffold. Furthermore, ALB-positive cells had also decreased. This result suggests that, at the last stage of liver regeneration, by the completion of replacement of a certain range of matrix near the borderline into normal live, the periportal ductular reaction was complete and hepatocytes could not engraft in the scaffolds and gradually declined in number. On the other hand, Ki67-positive cells and MMP2 -positive cells (Supplementary Fig. S1), considered to be fibroblasts, were observed on day 28, suggesting that remodeling of extracellular matrix and proliferation of hepatocytes were still occurring at this time point^28,29^.

In addition, to reveal infiltrated cellular function within the sutured scaffold, genetic analysis was performed. The genetic array result suggested the presence of hepatocytes, cholangiocytes and endothelial cells in the scaffold after implantation. Moreover, compared with healthy liver, sutured scaffold expressed similar genes associated with liver regeneration such as HGF, EGF and TGFβ. These findings suggest that the implanted scaffold promoted infiltration and migration of above cells and stimulate the damaged liver to accelerate liver regeneration. The reason MMP2 showed significantly higher gene expression in the scaffold is unclear, however, it might be considered that the sutured scaffold enhanced remodeling of extracellular matrix and contributed to liver regeneration. Providing adequate ECM for insulted liver might be able to enhance the liver regeneration after partial hepatectomy. Histochemical analysis showed the number of albumin positive cells was greater at day 10 than at day 28, while gene analysis showed that albumin mRNA expression was greater at day 28, suggesting the relative amount of albumin produced per cell increased. These results indicate that despite the small engraftment efficiency at day 28, engrafted hepatocytes had strong protein synthesis ability. Although, we need more number of analysis to reveal the mechanism of liver regeneration in our model, it is fact that the implanted scaffold promoted histological and functional liver regeneration. If other elements to make hepatocytes adherent were to be added, more effective liver regeneration might be expected.

Finally, the surgical procedures of liver resection and the implantation of the scaffold were performed successfully, without any complications, and pigs implanted with these scaffolds survived healthily for a month and did not develop obvious immunological problems^30^. From the view point of clinical applications, these surgical procedures, suturing the partial liver scaffolds onto the surfaces of partially hepatectomized livers, are considered simple and safe. Furthermore, sutured scaffolds can reduce exudates and bleeding at the resection site, which may cause biloma^31^, hematoma, or abscess, by covering the surface of the hepatectomized liver.

In conclusion, these results suggest that the implantation of a decellularized organ scaffold could promote structural reorganization after liver resection. This may increase the limit of liver resection volume and reduce surgical complications after liver surgery. However, the model did not demonstrate long-term regeneration of hepatocytes and bile duct structures. In addition, this study only examined the microscopic aspects of liver regeneration and did not evaluate clinical recovery after liver injury. In our experiment, we compared sacrificed liver volumes between control group and experimental group, however there was no difference. We consider the cause of this result is that the resected volume of the liver was not large enough to make a difference between two groups. In order to show clinical liver volume regeneration, it is necessary to conduct similar experiments and evaluation in a large liver resection model such as 60% hepatectomy porcine model.

Although, further experiments are required, this novel therapeutic strategy might be a viable surgical option to minimize the risk of post-operative liver failure.

## Materials and Methods

### Animals

All experimental procedures and protocols were approved by the Animal Ethics Committee of the Keio University, School of Medicine. The animals were treated according to the guidelines of the Ministry of Education, Culture, Sports, Science and Technology, Japan.

Male and female WLD pigs weighing 20–25 kg (Shiraishi Animals Co.,Ltd, Saitama, Japan) were used for liver harvesting for decellularization (n = 3) or partial hepatectomy (n = 3). Animals were anesthetized with midazolam 2 mg/body (Astellas, Tokyo, Japan) and medetomidine 0.08 mg/kg (Zenoaq, Fukushima, Japan), followed by isofluorane inhalation via a standard respiratory system, consisting of an endotracheal tube for continuing inhalation, to maintain anesthesia during the procedure.

All procedures involving animal use, housing and surgery were approved by performed in the fully equipped animal research laboratory located in the Laboratory Animal Center.

### Preparation of liver-derived ECM scaffolds

Harvesting of porcine liver was performed using the method described by Yagi *et al*.^12^. Our liver-derived ECM scaffolds were created by continuous detergent perfusion of entire dissected porcine livers. The frozen livers were thawed at 4 °C and subsequently washed with PBS to remove blood, through perfusion via the portal vein. Decellularization was achieved by perfusing the liver with sodium dodecyl sulfate in deionized water, followed by PBS with 1% Triton X, 0.05% EGTA, 0.05% sodium azide, and 4 mM CHAPS. The liver bioscaffold was washed extensively with sterile PBS and preserved in PBS supplemented with antibiotics, being kept at 4 °C for up to 7 days. The partial liver scaffolds were prepared by collecting the lower 1/3 of the left median or left lateral lobe of the decellularized porcine liver scaffold just before transplantation.

### Porcine model of liver regeneration with liver-derived ECM scaffolds

The pigs underwent laparotomy via a vertical midline incision. The left median lobe of the liver was used as the control lobe and the left lateral lobe was used as the treatment lobe. A clamp and crush method^32^ was used to avoid thermal damage to the resected surface for liver resection. Bleeding from the surface was sutured for hemostasis. Control lobes were partially hepatectomized without suturing any scaffolds, whereas, for the treatment lobes, the partial liver scaffolds were sutured onto the surfaces of similar sized, partially hepatectomized porcine livers, completely covering the area (Fig. 2a–c). After surgery, all animals were given unlimited access to food and water until the day of sacrifice.

### Histology and immunofluorescence analysis

Animals were sacrificed 1, 10 and 28 days after surgery and histological analysis was performed to evaluate the degree of cell infiltration into the sutured scaffolds. The qualitative and quantitative analysis of the infiltrated cells into the scaffolds was also assessed by immunofluorescence studies.

Decellularized liver matrix and liver samples at days 1, 10 and 28 were fixed with 10% formalin, embedded in paraffin, and processed for hematoxylin and eosin (H& E) staining. Additional samples at days 10 and 28 were permeabilized and incubated with anti-CD31, anti-albumin (ALB), anti-CK19, and anti-Epcam. The secondary antibodies were goat anti-rabbit IgG and goat anti-mouse IgG, and counter staining was performed with DAPI. We also performed double immunostaining of Ki67/ALB, Ki67/CK19, and Ki67/EpCAM to evaluate proliferation of cells appeared in the sutured scaffold. Quantification of positive areas was performed using the public software ImageJ. At days 10 and 28, albumin-positive cells and CD31-positive luminal structures were analyzed. The numbers of cells and luminal structures in five randomly selected areas per sample were counted in 20× images, using the public software ImageJ.

### Gene expression analysis

The harvested porcine samples were immediately submerged in RNAlater Stabilization Solution (ThermoFisher Scientific K.K., Tokyo, Japan) for gene expression analysis and stored at −20 °C until total RNA extraction. The samples containing from healthy liver and organ derived scaffold 10 or 28 days after implantation. The tissue was homogenized, and the total RNA was isolated using commercially available column-based purification kit (RNeasy Mini Kit, QIAGEN K.K. Tokyo, Japan) according to the manufacturer instruction, and the RNA content was measured with NanoDrop spectrophotometer (ThermoFisher Scientific K.K.). Complementary DNA (cDNA) was synthesized from 1 μg total RNA per samples using PrimeScript RT Master Mix (Takara Bio Inc., Shiga, Japan) according to the manufacturer instruction. Gene expression was analyzed using Power SYBR™ Green PCR Master Mix (ThermoFisher Scientific K.K.) and ViiA7 Real-Time PCR System (ThermoFisher Scientific K.K.) according to the manufacturer instruction. Gene list was presented in Supplementary Table S1. Obtained PCR data were analyzed by the comparative CT method. GAPDH was used as an internal control.

## Supplementary information

Supplementary information
