# Supplementary material for: Decellularized liver scaffolds promote liver regeneration after partial hepatectomy
Source: Sci Rep. 2019 Aug 29;9:12543. doi: 10.1038/s41598-019-48948-x (PMC6715632; doi:10.1038/s41598-019-48948-x)
Supplement: Supplementary file 1 — Supplementary information [file 41598_2019_48948_MOESM1_ESM.pdf]

Decellularized liver scaffolds promote liver regeneration after partial hepatectomy

Hirofumi Shimoda, MD, *Hiroshi Yagi, MD, PhD, FACS, Hisanobu Higashi, MD, Kazuki Tajima, DVM, PhD, Kohei Kuroda, DVM, PhD, Yuta Abe, MD, PhD, Minoru Kitago, MD, PhD, FACS, Masahiro Shinoda, MD, PhD, Yuko Kitagawa, MD, PhD, FACS,

Contents:

Supplementary Table S1

Supplementary Figure S1

Gene	Primer	Sequence
PECAM1	forward	TTCACAACGTCTCCTCCACG
	reverse	TTGGACACTCCTTCCACGAC
EpCAM	forward	CCCAACAAGGATGTGTGTGTG
	reverse	CTTCTCCCAGCCTTTGACCC
ALB	forward	AGAAGCAAACTGCACTCGTTG
	reverse	GTTGCCCAGGACAGTTCTCA
CYP27A1	forward	GCTGAGGAAGAACCAGACGG
	reverse	TGCTGGATCAGCCTTGTCAG
SOX17	forward	CTGAGCAAGATGCTGGGCAA
	reverse	GCGGCCGGTACTTGTAGTTG
EGF	forward	CGGATTTGCCCTGACCCTAC
	reverse	TCTCTGTGCTGACATCGCTC
HGF	forward	ACCATGCGAGGGAGATTATGG
	reverse	CCAGGACGATTTGGAATGGC
TGFβ1	forward	TTACAACAGTACCCGCGACC
	reverse	TAGATTTGGTTGCCGCTTTCC
MMP2	forward	GCAGTGATGGCAAGTTGTGG
	reverse	CAGGCTGTACCCTTGATCGG
GAPDH	forward	CACAGTCAAGGCGGAGAACGGGAA
	reverse	CTCCACAACATACGTAGCACCAGC

Supplementary Table. S1.

List of primers used for real-time PCR

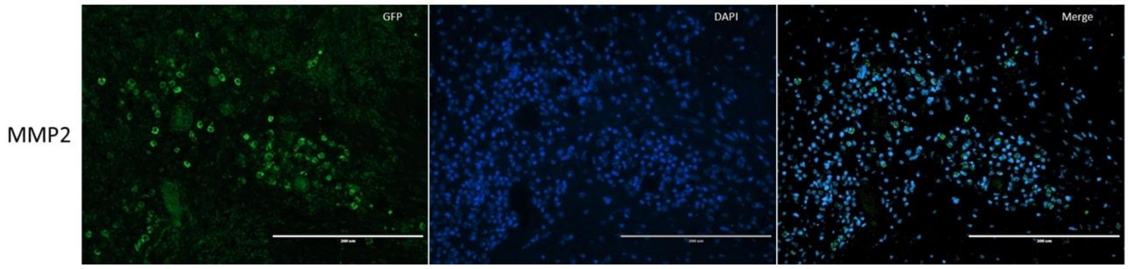

Supplementary Figure. S1.

MMP2 -positive cells, considered to be fibroblasts, were seen on day 28.
